# Companion Animals as Reservoirs of Multidrug Resistance—A Rare Case of an XDR, NDM-1-Producing *Pseudomonas aeruginosa* Strain of Feline Origin in Greece

**DOI:** 10.3390/vetsci12060576

**Published:** 2025-06-12

**Authors:** Marios Lysitsas, Eleftherios Triantafillou, Irene Chatzipanagiotidou, Anastasios Triantafillou, Georgia Agorou, Maria Eleni Filippitzi, Antonis Giakountis, George Valiakos

**Affiliations:** 1 Faculty of Veterinary Medicine, University of Thessaly, 43100 Karditsa, Greece; mlysitsas@uth.gr (M.L.); eichatzip@uth.gr (I.C.); anatriantafyllou@uth.gr (A.T.); 2 Vet Analyseis, Private Diagnostic Laboratory, 41335 Larissa, Greece; eltriantafil@gmail.com; 3 CeMIA SA, 41334 Larissa, Greece; gagorou@cemia.eu; 4 Faculty of Veterinary Medicine, Aristotle University of Thessaloniki, 54124 Thessaloniki, Greece; mefilippi@vet.auth.gr; 5 Department of Biochemistry and Biotechnology, University of Thessaly, 41500 Larissa, Greece; agiakountis@uth.gr

**Keywords:** *bla*_NDM-1_, carbapenemase, cat, *Pseudomonas aeruginosa*, ST308, virulence factors, WGS, XDR

## Abstract

A backyard cat with symptoms of otitis was transferred to a veterinary clinic in Central Greece. A sample was obtained and *P. aeruginosa* was isolated. The strain exhibited an extensively drug-resistant (XDR) profile, as it was non-susceptible to all tested agents except colistin. DNA extraction and whole-genome sequencing (WGS) were performed using a robotic extractor and Ion Torrent technology, respectively. The genome was assembled and screened for resistance and virulence determinants. The isolate belonged to the high-risk clone ST308 with a total of 67 antibiotic resistance genes (ARGs) and 221 virulence factor-related genes being identified. No plasmids were detected. The metallo-beta-lactamase (MBL) *bla*_NDM-1_ gene and 46 efflux pumps were included in the strain’s resistome. Both ARGs conferring tolerance to disinfecting agents and biofilm-related genes were identified, associated with the ability of this clone to adapt and persist in healthcare facilities. This case highlights the risk of relevant bacterial clones spreading in the community and even being transmitted to companion animals, causing challenging opportunistic infections to susceptible individuals, while others may become carriers, further spreading the clones to their owners, other animals and the environment.

## Simple Summary

*Pseudomonas aeruginosa* is a significant opportunistic pathogen in human and veterinary medicine. It commonly exhibits multidrug-resistant (MDR) profiles through an abundance of antibiotic resistance mechanisms. Because of its virulence and high adaptability in healthcare facilities, it is regularly implicated in challenging, severe infections, mostly in hospitalized patients. Several high-risk clones of *P. aeruginosa* are widely disseminated, raising concerns among healthcare professionals worldwide. In this study, an extensively drug-resistant (XDR) *P. aeruginosa* strain was isolated from a feline ear sample in Greece. Whole genome sequencing (WGS) was performed and the genome was assembled and submitted to identify antibiotic resistance genes (ARGs) and genes encoding virulence factors (VFs). The strain was typed as ST 308. A significant number of resistance determinants was identified, including numerous multidrug efflux pumps and the carbapenemase-producing gene *bla*_NDM-1_. Moreover, the plethora of detected VF-related genes highlighted the high virulence potential of the isolate. The results of this study indicate that high-risk bacterial clones can be disseminated in the community even outside the hospital environment. In this regard, companion animals could be infected by relevant strains, but also act as reservoirs, enhancing their further dissemination.

## 1. Introduction

*Pseudomonas aeruginosa* is a major opportunistic pathogen, included in the high-priority list of the World Health Organization (WHO) for bacterial pathogens of public health importance [1]. It is the etiologic agent of challenging healthcare-associated infections with high morbidity and mortality rates, especially in intensive care units [2].

Variable factors contribute to the introduction of *P. aeruginosa* in hospital environments and its establishment as a successful nosocomial pathogen. Its relatively large genome (5.5–7 Mbp) encodes several regulatory enzymes that allow *P. aeruginosa* to be highly adaptable to environmental changes [3]. Additionally, it possesses variable complex secretion systems that release toxins and enzymes such as proteases, elastases, and lipases, either to the extracellular environment or within the host cell [4], while its persistence in healthcare facilities and equipment is attributed to its ability to form mature, antibiotic-resistant biofilms.

*P. aeruginosa* is intrinsically resistant to variable antibiotics. The main mechanism to achieve this is its reduced outer membrane permeability, which is reported to be 12–100 times lower than that of *E. coli*, assisted by the intrinsic or induced expression of particular efflux pumps [5]. Moreover, it commonly acquires resistance to even more agents through mutational changes or horizontal gene transfer. Mobile genetic elements and mostly integrons play a critical role in the dissemination of resistance determinants among *P. aeruginosa* strains [3]. The acquisition of carbapenem resistance (CR) genes is of major clinical importance because it significantly reduces available therapeutic options. Strains carrying variable CR genes have been detected worldwide, including metallo-β-lactamases (MBL) producing *P. aeruginosa* [6]. These enzymes efficiently hydrolyze all β-lactam antibiotics except monobactams, even when combined with β-lactamase inhibitors. Several MBLs have been reported, including the SPM-, GIM-, SIM-, KHM-, NDM-, AIM-, DIM-, SMB-, TMB-, and FIM-type enzymes [7]. Among them, New Delhi metallo-β-lactamase (NDM) is a worldwide distributed variant, identified in over 60 different bacterial species, with the highest prevalence documented in the Indian subcontinent, the Middle East, and the Balkans [8].

Moreover, *P. aeruginosa* clones regularly exhibit a decreased susceptibility to disinfectants and antiseptics. In particular, efflux-pump-mediated tolerance towards different classes of biocides, such as phenolic compounds, alkylating agents, cationic biocides, and oxidizing compounds, has been well documented in the literature [9]. This characteristic, combined with its ability to form strong biofilms on a great variety of biotic and abiotic surfaces, prevents its effective eradication from healthcare facilities and equipment.

In companion animals, *P. aeruginosa* is regularly implicated in soft tissue infections (pyoderma, wound infection, etc.), chronic otitis externa, ocular infections such as ulcerative keratitis, and respiratory and urinary tract infections [10]. The treatment of relevant cases is challenging because the available therapeutic options for use against *P. aeruginosa* in pets are limited and the acquisition of resistance to even more antibiotics by the isolates is common [11,12].

WGS technologies are increasingly being evaluated in AMR research globally, as they constitute key techniques for rapidly and effectively understanding pathogen evolution, population dynamics, and genomic epidemiology [13,14]. Several recent studies have investigated the variable characteristics of *P. aeruginosa* isolates using sequencing approaches [15,16,17,18,19].

The objectives of this study are to present a rare case of an XDR *P. aeruginosa* strain obtained from the ear of a backyard cat in Greece and analyze the phenotypic and molecular characteristics of this isolate, focusing on antimicrobial resistance (AMR) and virulence aspects of major importance. Finally, we highlight the emerging threat of the distribution of high-risk bacterial clones in the community and the potential of companion animals to act as reservoirs for relevant strains and contribute to their further dissemination.

## 2. Materials and Methods

### 2.1. Case Presentation

A backyard cat was transferred to a veterinary clinic in Volos, Greece due to head shaking and ear scratching. Erythema and unilateral purulent auricular discharge were observed during the initial examination. During clinical investigation, a specimen was obtained for aerobic culture. A sample of ear secretions was aseptically collected using a sterile swab from the horizontal portion of the external ear canal. It was immediately placed in Stuart’s transport medium and transferred to the laboratory. The swab was inoculated onto 90 mm Petri dishes with sheep blood agar, MacConkey agar, and mannitol salt agar (Oxoid, Hampshire, UK). The plates were then incubated at 37 °C for 24 h. Growth was detected on sheep blood and MacConkey agar plates. Gray to green metallic colonies surrounded by a narrow zone of beta-hemolysis on blood agar and a positive oxidase reaction were observed. *Pseudomonas aeruginosa* was identified using a commercial identification kit, according to the manufacturer’s instructions (Microgen GN-ID A+B, Gold Standard Diagnostics, Budapest, Hungary). The strain was subjected to routine susceptibility testing using the disc diffusion method and exhibited a resistance profile against all tested agents, particularly ceftazidime, cefepime, piperacillin-tazobactam, ticarcillin-clavulanate, aztreonam, imipenem, meropenem, amikacin, gentamicin, tobramycin, ciprofloxacin, enrofloxacin, and fosfomycin. The resistance profile was then confirmed using the minimum inhibitory concentration (MIC) method (VITEK^®^2, bioMérieux, Craponne, France). The isolate was also resistant to ceftazidime–avibactam, ceftolozane–tazobactam, imipenem–relabactam, meropenem–vaborbactam, and fosfomycin, but was susceptible to colistin (MIC = 1 μg/mL) and intermediate to aztreonam (MIC = 16 μg/mL) (Table 1). Considering the agents tested and the susceptibility profile, this strain can be classified as XDR according to previously defined criteria [20].

### 2.2. Nucleic Acid Extraction and Whole Genome Sequencing

Pure colonies of the strain were inoculated in plates of a general-purpose medium (Nutrient Agar, Oxoid, Hampshire, UK) and incubated overnight at 37 °C. Whole DNA was extracted from a selected pure colony using an IDEAL™ 32 extraction robot (Innovative Diagnostics, Grabels, France) and a compatible commercial kit (ID GeneTM Mag Fast Extraction Kit, Innovative Diagnostics, Grabels, France), according to the manufacturer’s instructions. A fluorometer (Invitrogen QubitTM 4, Thermo Fisher Scientific, Waltham, MA, USA) was used to measure the DNA concentration in the obtained extract.

A DNA library was prepared from 100 ng of gDNA using the Ion Xpress Plus Fragment Library Kit (Thermo Scientific, Waltham, MA, USA). The library was indexed with a unique adapter using the Ion Xpress barcode adapter kit (Thermo Scientific, Waltham, MA, USA). The barcoded library was purified using the Agencourt AMPure XP Beads (Beckman Coulter, Brea, CA, USA), quantified with a Qubit 2.0 fluorometer (Thermo Scientific, Waltham, MA, USA), and diluted to 100 pM. Template preparation, enrichment, and chip loading were performed using an Ion Chef system (Thermo Scientific, Waltham, MA, USA). Sequencing was performed on the S5XL instrument with a 530 chip using the Ion 510 & Ion 520 & Ion 530 Kit–Chef (Thermo Scientific, Waltham, MA, USA). All procedures were performed according to the manufacturer’s instructions. Base calling and demultiplexing of the raw sequencing data were performed in the Torrent Suite 5.10 software (Thermo Scientific, Waltham, MA, USA) using the default parameters.

### 2.3. Sequencing Analysis

The sequencing data were uploaded to the Galaxy web platform, and the server at usegalaxy.org was used to analyze the data [23]. The quality of raw reads was assessed using FastQC v0.12.1 [24]. A total of 2,995,002 sequences and 1 Gbp bases were identified, while there were no poor-quality flagged sequences. Subsequently, the reads were trimmed using Sickle software (v1.33.3) [25]. The draft genome was assembled de novo into contigs using SPAdes v3.15.5 [26,27,28,29]. Assembly metrics were calculated using Quast v5.3.0. [30,31] and contigs shorter than 200 bp were excluded from the downstream analysis. The strain was typed using MLST v2.22.0 [32]. For the detection of plasmids and ARGs, staramr (v0.11.0) was initially utilized [33], while contigs were screened for previously characterized virulence genes with ABRicate v1.0.1 (threshold: 95% identity, 90% coverage) [34]. Finally, the assembled genome file was uploaded to the Comprehensive Antibiotic Resistance Database (CARD v4.0.0) web portal [35] to detect ARGs, including multidrug efflux pumps (threshold: 95% identity, 90% coverage). Genomic maps were visualized using Proksee v2.0 [36]. BLAST v.1.4.1 webtool [37] was used for genome comparison with NDM-1-producing *P. aeruginosa* strains. Computation of whole genome Average Nucleotide Identity was performed using FastANI v1.34 [38]. The genome sequence was annotated using Bakta v.1.8.2 [39] and Prokka v1.14.6 [40]. Mobile genetic elements (MGEs) were detected using mobileOG-db (beatrix-1.6) software [41].

The genome was deposited in GenBank under the accession number JBMPIE000000000 (BioProject accession: PRJNA1244906, BioSample: SAMN47734949).

Sankey diagram for VF-related genes was created with sankeyMATIC (https://sankeymatic.com/, accessed on 8 May 2025).

## 3. Results

The size of the assembled genome was 6.96 Mbp, with a G+C content of 65.96%. According to multilocus sequence typing (MLST), the strain belonged to ST 308, which is considered a high-risk international clone.

A total of 71 ARGs were identified in CARD and 67 of them were included in the analysis, according to the applied thresholds. Four genes were excluded from further analysis because of low identity of the matching region (61.1%, 43.8%, 41.2%, and 32.3%, respectively). No plasmid was detected. The respective genes and the anticipated phenotypic resistance are presented in Table 2.

**Figure 1:**
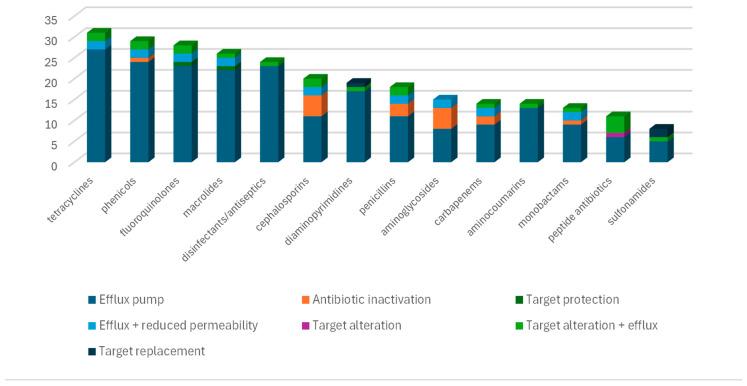
Three-dimensional stacked-column chart presenting the number of ARGs per category of resistance mechanism and per class of antibacterial agents.

The results of the susceptibility testing were confirmed by the strain’s resistome. All available antibiotic classes for usage against *Pseudomonas aeruginosa* in veterinary medicine were entailed in the predicted phenotype, apart from polymyxins. The same has been applied for critical antibiotics for hospital-acquired infections in humans, such as carbapenems and fosfomycin. A great number of multidrug efflux pumps were identified, conferring variable levels of resistance against several classes of antibiotics. Moreover, 23 of these efflux pump genes are associated with increased tolerance to disinfecting agents and antiseptics (Figure 1). Finally, ARGs producing inactivating enzymes were detected for aminoglycosides, β-lactams and phenicol antibiotics, including the carbapenemase-producing gene *bla*_NDM-1_. The detailed data on the identified ARGs are presented in Table S1.

A total of 31 genes conferring resistance to tetracyclines were identified, and numerous ARGs conferring resistance to phenicols, fluoroquinolones, and macrolides were also detected. Most of these genes encode multidrug efflux pumps. Tolerance to disinfecting agents and antiseptics is also mediated mostly by pump genes. In contrast, antibiotic-inactivating genes were mostly related to beta-lactams and aminoglycosides, apart from the chloramphenicol acetyltransferase *catB7*. Three target replacement genes for sulfonamide and diaminopyrimidine antibiotics were detected, whereas target protection and target alteration mechanisms were documented in one ARG for fluoroquinolones (*qnr*_VC1_), macrolides (*msr*E), and peptide antibiotics (*arn*A).

**Figure 2:**
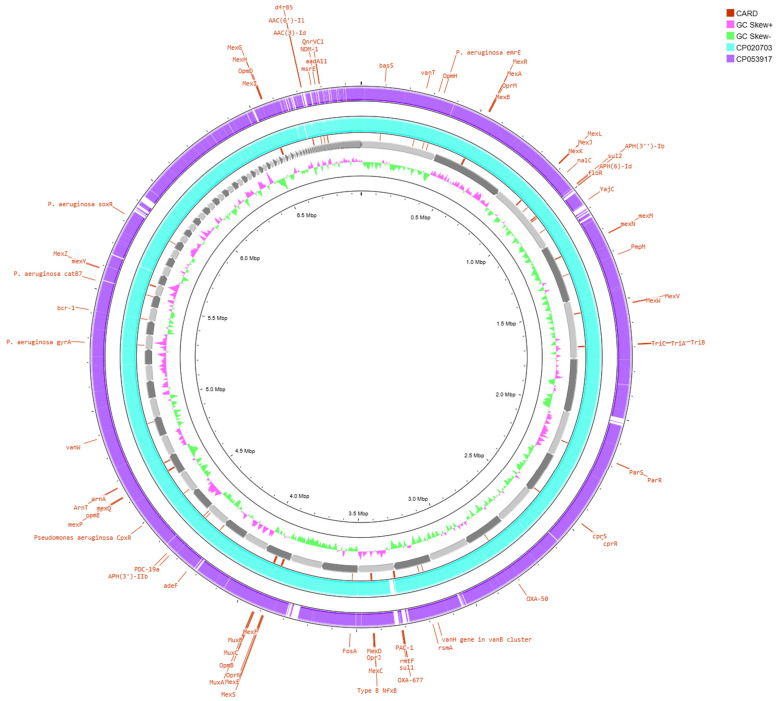
The circular map of the genome of the *P. aeruginosa* isolate illustrating key genetic features, including resistance determinants such as *bla*_NDM-1_. The innermost circle illustrates the genome position, followed by a ring presenting the GC skewness. The next ring illustrates the contigs, whereas the subsequent two rings represent the presence or absence of BLAST matches at specific positions, with each ring corresponding to the WGS of the indicative NDM-1-producing *P. aeruginosa* strains (CP020703: ST308 strain isolated in Singapore [42] and CP053917: ST773 strain isolated in South Korea [43]). Regions covered by BLAST alignments are presented by solid colors, while white spaces denote genomic areas not covered by the alignments (created in https://proksee.ca/, accessed on 2 April 2025, the respective JSON file is provided as Supplementary File S1).

BLAST comparison results revealed a close genetic relationship between the isolate analyzed in this study and the NDM-1-producing ST308 clone previously reported in Singapore [42], since limited regions of difference (RODs) were identified (Figure 2). A summary of the Average Nucleotide Identity (ANI) values between the sequenced strain and the two reference genomes is provided in Table 3, and detailed BLAST alignment data are available in Supplementary Table S2.

**Figure 3:**
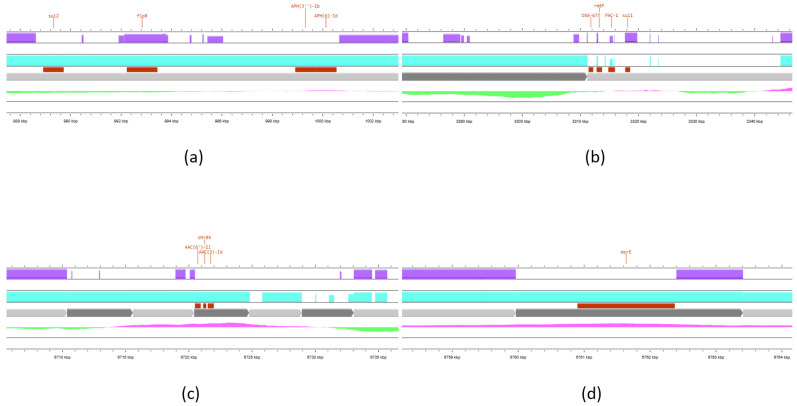
Focused regions of genomic variability extracted from the map shown in Figure 2. Solid-colored areas represent regions aligned by BLAST, whereas white gaps indicate regions lacking alignment coverage. Panels (**a**,**c**,**d**) highlight the highly conserved regions containing ARGs between the sequenced strain and *P. aeruginosa* strain CP020703, with notable divergence observed in strain CP053917. Panel (**b**) shows ARGs *bla*_OXA-677_, *rmt*F, *bla*_PAC-1_, and *sul*1 located within a region that displays substantial variability across all compared genomes (map created using https://proksee.ca/, accessed on 13 May 2025).

As shown in Table 3, a high degree of sequence conservation was observed, particularly in comparison to the *P. aeruginosa* ST308 Singapore strain (CP020703) [42]. In reference to the strain’s resistome, genomic map analysis revealed that the majority of ARGs, including *bla*_NDM-1,_ are located within highly conserved genomic regions shared across all three strains. In contrast, only a limited number of ARGs was found within the variable regions, as illustrated in Figure 3.

ARGs presented in Figure 3 were mostly found within regions conserved in both ST308 strains, but were absent or disrupted in strain CP053917. However, one region of interest (panel b) harbored multiple ARGs within a locus, exhibiting substantial variability. By adding the identified MGEs to the genomic map (Figure S1), it is indicated that all four non-conserved genomic regions where ARGs are located also involve several elements such as prophage regions, recombination/repair protein genes, conjugative transfer genes, and integration/excision modules. These MGEs can facilitate nucleic acid recombination and the acquisition of foreign DNA in these regions, thereby enhancing genetic divergence.

The annotated bacterial genome is shown in Figure S2, and a summary of annotations from the Bakta and Prokka tools is provided in Table S3.

**Figure 4:**
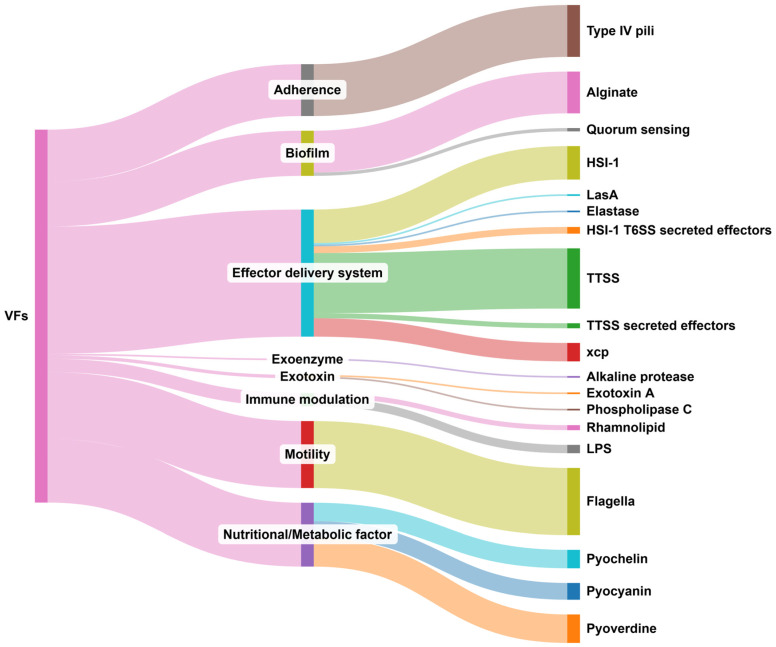
Sankey diagram presenting the distribution of VF-related genes identified with ABRicate per mechanism of virulence and major VF. Genes are classified according to the relevant VF category (middle column) and further classified according to the major VF they mediate (left column).

In reference to the strain’s virulence, a total of 221 VF-related genes were detected. All of these genes encode previously characterized virulence factors that are regularly involved in the virulome of *P. aeruginosa*. The detailed data are presented in Table S4. The distribution of these genes according to the relevant classification of genes provided in the virulence factor database (VFDB) [44] is presented in Figure 4.

A total of 75 genes belong to effector delivery systems, with the majority of them associated with the Type III secretion system and the correlated secreted effectors. Another group of 40 genes is related to flagella-mediated motility of the strain. Thirty-seven genes encode nutritional/metabolic factors, specifically pyochelin, pyocyanin, and pyoverdine, which mediate bacterial metabolic pathways such as iron acquisition and cytotoxicity. A total of 31 genes are associated with adherence. Type IV pili genes that mediate host cell attachment and motility along the cell membrane were exclusively identified. A total of 27 genes that contribute to biofilm production were identified. Most of these are alginate biosynthetic genes, while two quorum sensing system genes, crucial for mature biofilm formation, are also present. Finally, eight genes are relevant to immune modulation, two encode exotoxins and one encodes an exoenzyme (alkaline protease).

## 4. Discussion

This study highlights the wide distribution of high-risk MDR clones in the community and the potential of pets to act as reservoirs for relevant strains, which can be further disseminated to their environment, including other animals or their owners. To our knowledge, this is the first report of both XDR- and *bla*_NDM_-positive *P. aeruginosa* of animal origin in the country. Additionally, to our knowledge, this is the first report of XDR- and *bla*_NDM_-positive *P. aeruginosa* of feline origin in the current literature, as the limited relevant references mostly concern livestock animals [45].

Obtaining a strain exhibiting a multidrug-resistant profile from a site of potential infection constitutes a challenge for veterinarians because of the lack of available therapeutic options. However, in our case, isolation of this strain after aerobic culture of the ear specimen did not clarify the exact etiology of otitis. In addition, bacteria are mostly reported to be perpetuating and are not primary factors of otitis in cats [46,47]. Therefore, it is possible that the isolate colonized the ear and other factors were responsible for disease progression. However, the presence of a high-risk clone, even as part of the normal flora, is undoubtedly concerning, and should be treated with caution.

The strain was typed as ST308. Through evolutionary changes in its genome [48] and persistence in variable environments [49], it has become a successful high-risk clone for healthcare facilities worldwide [42]. It has occasionally been associated with different MBLs, particularly the IMP type [50], in contrast to the isolate in this study. However, NDM-1 producing ST308 strains have recently been reported in Southeast Asia [42,43,51]. The results of BLAST comparison indicated a close relationship between this feline isolate and the high-risk clone reported in Singapore [42]. Interestingly, recent studies in Greece have also highlighted the emergence of *bla*_NDM-1_-positive *P. aeruginosa* ST308 in hospital environments in the country [18,19], including central Greece. In reference to companion animals, an IMP-45-producing *P. aeruginosa* ST308 strain was isolated from a canine sample in China [52]; however, the implication of *P. aeruginosa* ST308 in feline infections has not yet been explored.

The strain exhibited an XDR resistance profile. Studies on XDR resistance profiles in isolates from pets are limited [53,54]. It was phenotypically resistant to imipenem, meropenem, and their combination with relabactam and vaborbactam, respectively. These AST results were confirmed by the detection of *bla*_NDM-1_ gene. This is a critical finding since reports on carbapenem-resistant bacteria from companion animals are limited. However, their incidence is rather increasing worldwide. Canine and feline strains of *P. aeruginosa* have been reported to exhibit phenotypic resistance to carbapenems [55,56,57,58] or carry carbapenemase-producing genes such as *bla*_VIM_ [59,60], *bla*_IMP_ [52], *bla*_KPC_ [61], and combinations of *bla*_OXA,_ *bla*_PAO_, and *bla*_PDC_ genes [58]. However, the MBL-producing gene *bla*_NDM_ has been identified mainly in canine and feline Enterobacteriaceae isolates [53,62,63,64] and not in *P. aeruginosa*.

Among the identified ARGs, the presence of *bla*_PDC-19a_ is also noteworthy, as it confers resistance not only to carbapenems but also to aztreonam, an agent that is often considered a potential therapeutic option against MBL-producing strains [65]. Recent studies have reported that the *bla*_PDC-19a_ variant is detected exclusively in carbapenem-resistant [66] and ST308 [67] *P. aeruginosa* isolates, which is consistent with the results of the present study. However, references for its detection in strains from companion animals are scarce [60]. Notably, a global increase in the prevalence of *P. aeruginosa* carrying *bla*_PDC-19a_ has been documented over the last few decades, with this variant rising from the tenth most frequently detected between 2000 and 2012 to the third most common between 2022 and 2024 [68].

Additionally, 46 efflux pump genes from five distinct families (or superfamilies) were identified in the resistome of the strain (Table S1). Most belong to the resistance/nodulation/cell division (RND) family, representing the primary efflux system involved in antibiotic resistance in *P. aeruginosa.* These pumps also contribute to biofilm formation, thereby enhancing the persistence and virulence of the pathogen [69]. The presence of genes conferring resistance to multiple classes of antibiotics could simultaneously affect AMR dynamics in veterinary settings, as resistance to critical antibacterial agents could be co-selected under the selective pressure of commonly used antimicrobials in pets. These findings align with those of previous studies, where the pivotal role of efflux mechanisms in the AMR of strains from pets was highlighted [60,70].

Furthermore, 23 genes that confer tolerance to antiseptics and disinfectants were identified in the sequenced strain. These genes represent critical factors for the persistence of nosocomial strains within healthcare environments and their subsequent dissemination through medical equipment, personnel, and infected patients [9]. Biocide resistance among *P. aeruginosa* isolates from companion animals has been previously reported [71,72]. Considering the co-occurrence of biocide tolerance and biofilm-producing determinants in relevant “successful” nosocomial strains, the risk of their dissemination and long-term establishment in veterinary facilities cannot be overlooked. This concern is reinforced by the fact that veterinary clinics exhibit environmental and operational similarities to human hospitals, including a variety of available biotic and abiotic surfaces, frequent handling of patients, and selective pressure imposed by the administered antibiotics.

BLAST comparison (Table 3 and Table S3) demonstrated a high degree of genetic similarity between the sequenced strain and a high-risk clone associated with human infections in Singapore [42], highlighting the potential for the international spread of such clinically significant clones. This finding is particularly concerning considering that similar strains have been reported in both human and companion animal infections, emphasizing the need for a One Health approach in the epidemiological monitoring and control of *P. aeruginosa* [73]. Furthermore, genomic map analysis revealed that in non-conserved genomic regions identified in our *P. aeruginosa* strain, compared to the two literature strains, the presence of MGE-related sequences such as prophages, genes encoding recombination/repair proteins, conjugative transfer genes, and integration/excision modules is regular. These regions likely represent genomic islands or integrative elements that facilitate the acquisition of foreign DNA, thereby contributing to the adaptive potential of the bacterium. These elements are well-documented in *P. aeruginosa.* Its genome has been described as a mosaic structure of a highly conserved core genome interrupted by elements inserted into the chromosome at various loci, constituting the “accessory genome”, which has been associated with increased virulence, antibiotic resistance, and environmental persistence [74,75]. Therefore, these non-conserved regions may not simply reflect random divergence but could confer functional advantages that promote the pathogenicity and resilience of the strain. Understanding the content and potential functions of these regions is crucial for elucidating the mechanisms of bacterial adaptation and development of resistance.

The presence of VFs identified in the genome of the sequenced isolate (Table S2) demonstrated its pathogenic potential. Factors such as Exotoxin A as well as type III and VI secretion systems have been correlated with nosocomial strains and multidrug resistance in the literature [2,76], and were unsurprisingly identified in previous studies of NDM-1-positive *P. aeruginosa* ST 308 strains [18,19,42]. Moreover, the presence of *exo*U gene, which encodes a secreted effector protein, has been associated with poor prognosis in human infections [2,77] and has been reported in high-risk *P. aeruginosa* canine isolates [56]. In contrast, it was rarely detected in pet-associated strains in Algeria [58] and was absent in a feline strain in Mexico [78]. In another study, *nan*1 and *fli*C genes were correlated with mature biofilm formation and increased virulence in canine and feline isolates [79]. However, these genes were not identified in this study. Similarly, among the five most commonly identified virulence genes in feline isolates from Poland [80], only two, *las*B and *plc*H, were present in the current strain. These findings underscore the considerable variability in the virulence gene profiles of feline *P. aeruginosa* isolates reported in the literature, even though the current data are rather limited. Therefore, it remains challenging to determine which specific VFs contribute to inflammation in the feline ear canal. Biofilm formation is undoubtedly a major factor correlated with chronic infections and poor outcomes in cases of otitis in pets, while the role of bacterial proteases, such as alkaline protease and elastases A and B, is also expected to be significant [81]. Moreover, their inhibition exhibited encouraging results in canine otitis [82]. Finally, exotoxin production is likely crucial for the progression of inflammation, primarily by enhancing cytotoxicity, resulting in phagocyte killing and disruption of the epithelial barriers [83]. Combining sequencing results with phenotypic assays and gene expression profiling could provide more comprehensive insights into the virulence potential of the isolate.

## 5. Conclusions

In conclusion, the detection of an MBL-producing isolate from a backyard companion animal raises concerns about the potential distribution of high-risk bacterial clones or associated molecular determinants in the community. This issue is particularly troubling, given the lack of established official surveillance systems for pets; therefore, the actual prevalence of such resistance determinants may be underestimated. Furthermore, there is growing evidence that the microbiota of pets and their owners are altered by close contact [84]. In this regard, the transmission of MDR strains from owners to animals and vice versa is substantial [85]. This is of great importance in the context of a global rise in reported infections caused by MBL-producing strains, which are frequently associated with high mortality rates [86]. Moreover, there is a significant risk of nosocomial pathogen transmission and hospital-acquired infections in veterinary clinical settings [87]. Accordingly, the presence of an XDR high-risk clone in an infected pet not only represents a therapeutic challenge for veterinary professionals but also raises considerable public health concerns.

From a future perspective, preventive and surveillance strategies targeting AMR at the community level should include companion animals and veterinary healthcare settings. Veterinarians should be informed, vigilant, and adhere to the principles of prudent antimicrobial usage. Effective mitigation of the phenomenon requires a multidisciplinary approach—one that integrates human, animal, and environmental health, along with their dynamic interactions, in alignment with the One Health framework.

## Figures and Tables

**Table 1:** Antibacterial agents, disc content, breakpoints used in this study [21,22] and AST results.

Antibacterial Agent	Disk Content (μg)	Breakpoints		AST Results	
		Inhibition Zone (mm)	MIC μg/mL	DD	MIC
Ceftazidime	30	S: ≥18 I: 15–17, R: ≤14	S: ≤8, I: 16, R: ≥32	R	R
Cefepime	30	S: ≥18 I: 15–17, R: ≤14	S: ≤8, I: 16, R: ≥32	R	R
Piperacillin–tazobactam	100/10	S: ≥22 I: 18–21, R: ≤17	S: ≤16/4, I: 32/4, R: ≥64/4	R	R
Ticarcillin–clavulanate	75/10	S: ≥24 I: 16–23, R: ≤15	-	R	NT
Aztreonam	30	S: ≥22 I: 16–21, R: ≤15	S: ≤8, I: 16, R: ≥32	R	I
Imipenem	10	S: ≥19 I: 16–18, R: ≤15	S: ≤2, I: 4, R: ≥8	R	R
Meropenem	10	S: ≥19 I: 16–18, R: ≤15	S: ≤2, I: 4, R: ≥8	R	R
Ceftazidime–avibactam	-	-	S: ≤8/4, R: ≥16/4	NT	R
Ceftolozane–tazobactam	-	-	S: ≤4/4, I: 8/4, R: ≥16/4	NT	R
Imipenem–relabactam	-	-	S: ≤2/4, I: 4/4, R: ≥8/4	NT	R
Meropenem + vaborbactam	-	-	S: ≤16/4, I: 32/4, R: ≥64/4	NT	R
Amikacin	30	S: ≥17 I: 15–16, R: ≤14	S: ≤16, I: 32, R: ≥64	R	R
Gentamicin	10	S: ≥16 I: 13–14, R: ≤12	S: ≤2, I: 4, R: ≥8	R	R
Tobramycin	10	S: ≥19 I: 13–18, R: ≤12	S: ≤1, I: 2, R: ≥4	R	R
Ciprofloxacin	5	S: ≥25 I: 19–24, R: ≤18	S: ≤0.5, I: 1, R: ≥2	R	R
Enrofloxacin	5	S: ≥23 I: 17–22, R: ≤16	S: ≤0.5, I: 1–2, R: ≥4	R	R
Fosfomycin ^1^	200	-	-	R	R
Colistin	-	-	S: ≤2, R: ≥4	NT	S

^1^ Since there are no available breakpoints, the AST result for fosfomycin (resistant) was interpreted empirically, based on the total absence of an inhibition zone and an MIC ≥ 256. Abbreviations: AST: antibiotic susceptibility testing, DD: disc diffusion, MIC: minimum inhibitory concentration, NT: not tested, R: resistant, I: intermediate, S: susceptible.

**Table 2:** Identified resistance genes and the respective predicted phenotype.

Resistance Gene	Predicted Phenotype
*aac(6′)-Il*, *aadA11*, *aph(3″)-Ib*, *aph(3′)-IIb*, *aph(6)-Id*, *EmrE*	amikacin, gentamicin, kanamycin, neomycin, spectinomycin, streptomycin, tobramycin
*bla*_NDM-1_*, bla*_PDC-19a_, *bla*_PAC-1_, *bla*_OXA-677_, *bla*_OXA-50_	all beta-lactams except monobactams
*catB7*	chloramphenicol
*floR*, *mexM*, *mexN*	florfenicol
*qnrVC1*	ciprofloxacin
*fosA*	fosfomycin
*msrE*	erythromycin, azithromycin
*dfrB5*	trimethoprim
*sul1*, *sul2*	sulfamethoxazole
*cprR*, *cprS*, *basS*, *arnA*	peptide antibiotic
*MexA*, *OprM*, *YajC*, *rsmA*, *MexE*, *MexF*, *OpmB*, *MuxC*, *MuxB*, *CpxR*, *MexB*, *MexK*, *MexJ*, *MexL*, *PmpM*, *MexV*, *MexW*, *MexC*, *MexD*, *OprJ*, *OprN*, *MuxA*, *opmE*, *mexQ*, *mexP*, *MexG*, *MexH*, *MexI*, *OpmD*, *nalC*, Type A *NfxB*, *MexS*, *MexZ*, *ParS*, *ParR*, *soxR*, *MexR*	aminocoumarins, aminoglycosides, beta-lactams, diaminopyrimidines, fluoroquinolones, macrolides, tetracyclines, oxazolidinones, peptide antibiotics, phenicols, rifamycin, sulfonamides, disinfecting agents and antiseptics (multidrug efflux pumps)
*TriA*, *TriB*, *TriC*, *OpmH*	disinfecting agents and antiseptics

**Table 3:** Summary results of BLAST comparison between the sequenced strain and bla_NDM_-1 *P. aeruginosa* strains.

Metric	CP053917_vs_Sequenced_Strain	CP020703_vs_Sequenced_Strain
Average Nucleotide Identity (ANI)	99.094	99.9328
Query Sequence Fragments	2308	2297
Orthologous Matches	2064	2188
% orthology	89.4	95.3

## Data Availability

The original genomic data presented in this study are openly available in GenBank under the accession number JBMPIE000000000 (BioProject accession: PRJNA1244906, BioSample: SAMN47734949).

## Supplementary Materials

The following supporting information can be downloaded at: https://www.mdpi.com/article/10.3390/vetsci12060576/s1, Table S1: ARGs identified in CARD; Table S2: Summary of BLAST results; Table S3: Summary of annotations; Table S4: VF-related genes, Figure S1: Panels from Figure 3 including annotated MGEs, Figure S2: Map of annotated bacterial genome; File S1: JSON file from the genomic map of Figure 2.
