# Supplementary material for: Companion Animals as Reservoirs of Multidrug Resistance—A Rare Case of an XDR, NDM-1-Producing Pseudomonas aeruginosa Strain of Feline Origin in Greece
Source: Vet Sci. 2025 Jun 12;12(6):576. doi: 10.3390/vetsci12060576 (PMC12197500; doi:10.3390/vetsci12060576)
Supplement: Supplementary file 1 [file vetsci-12-00576-s001.zip › Supplementary data/Figure S1.pdf]

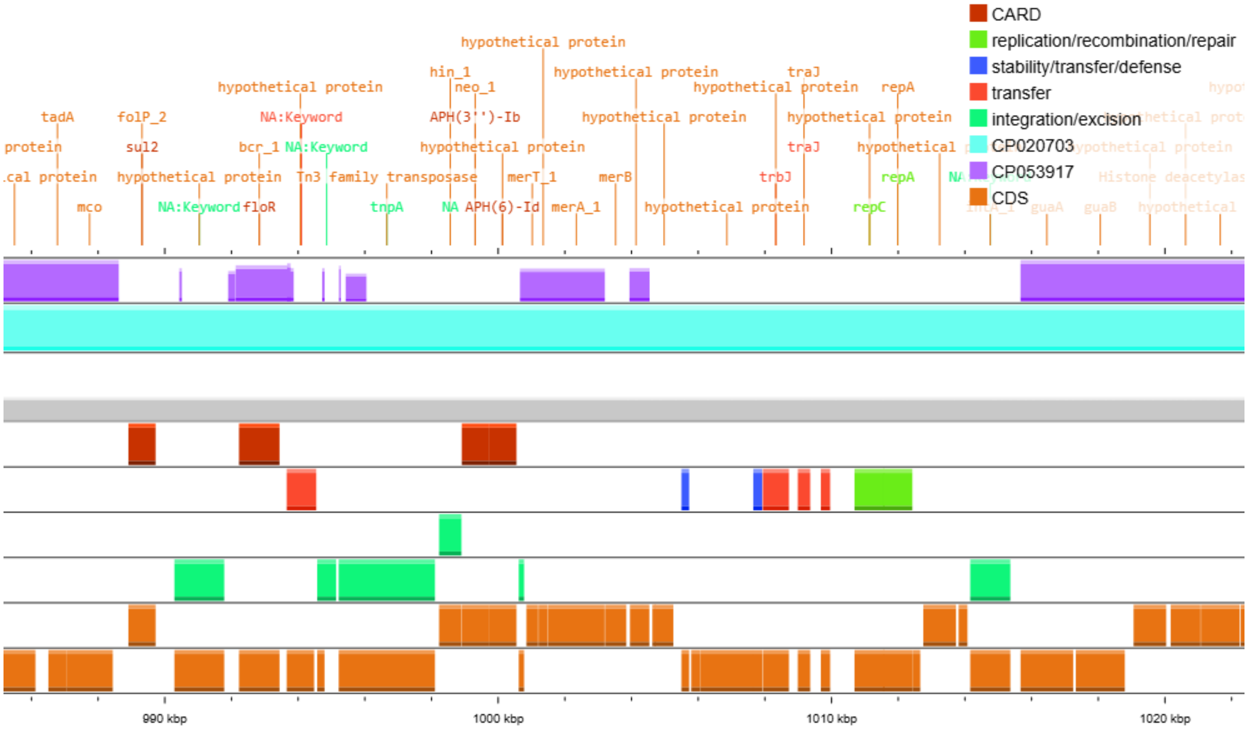

(a)

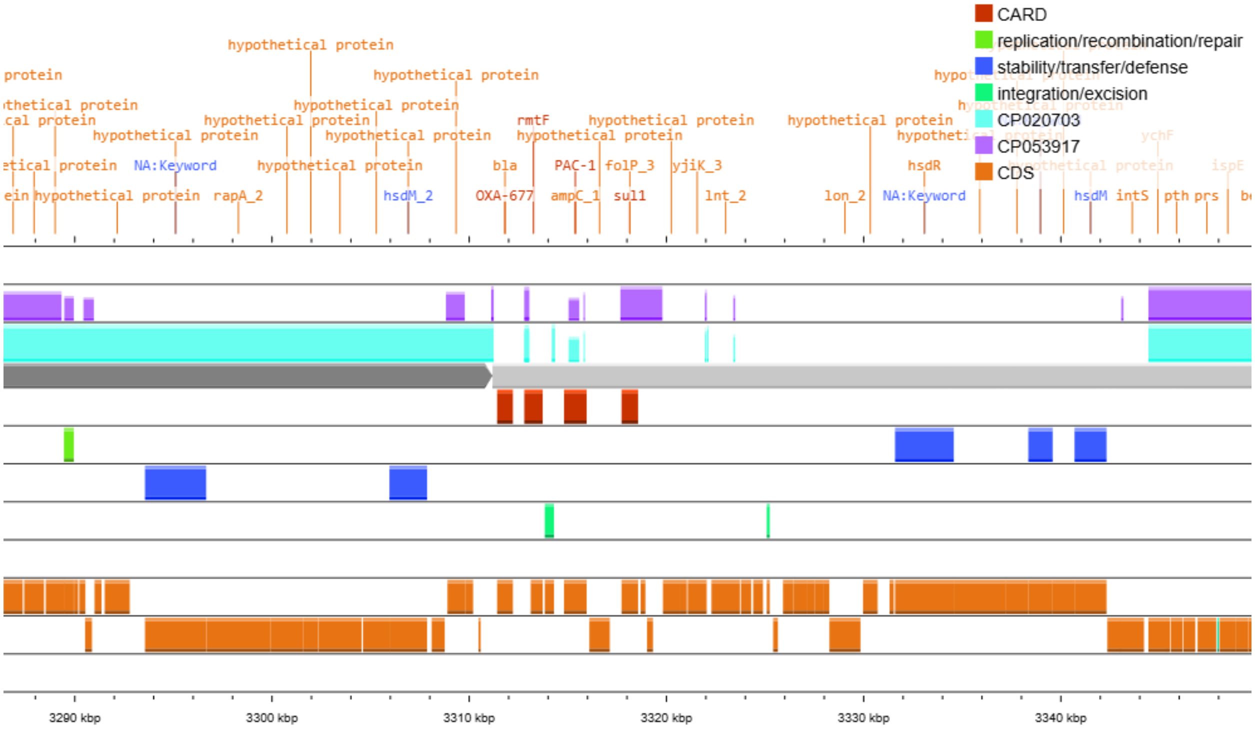

(b)

(a), (b), (c), (d). The respective focused regions of Figure 3 including identified MGEs such as prophage regions, recombination/repair protein genes, conjugative transfer genes, and integration/excision modules (Map created using https://proksee.ca/, accessed on 13 May 2025)

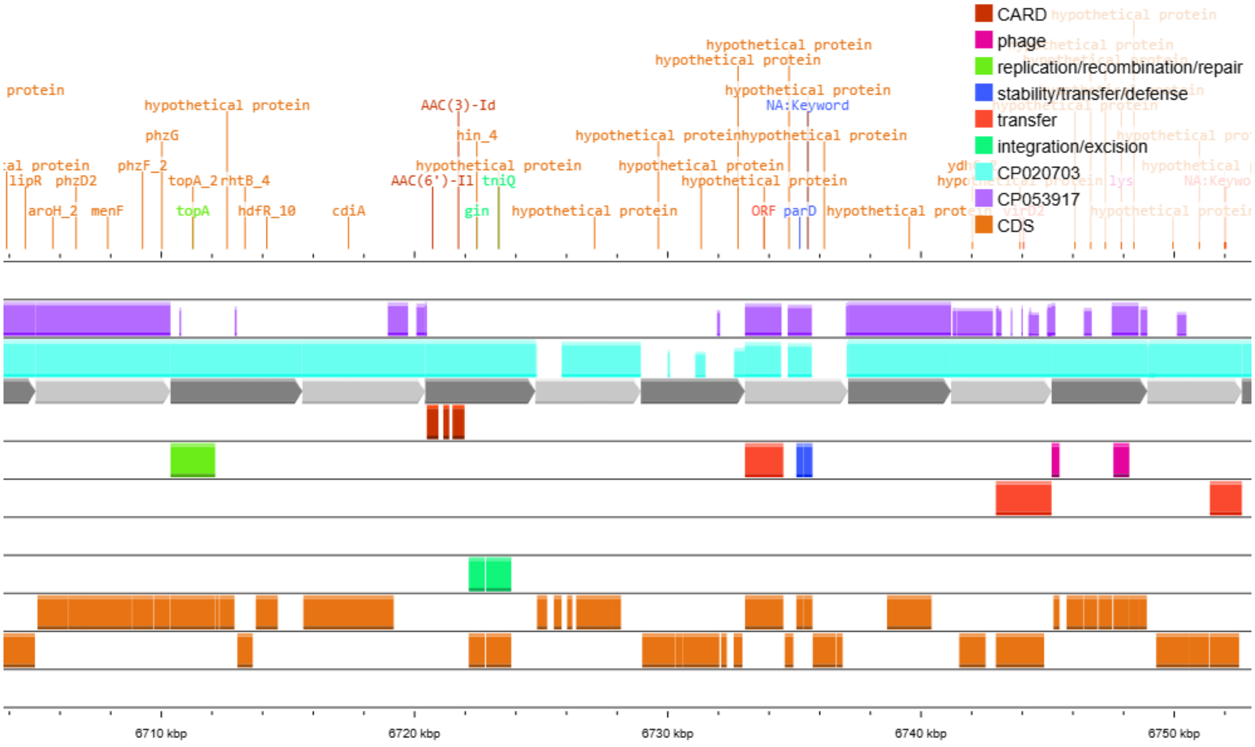

(c)

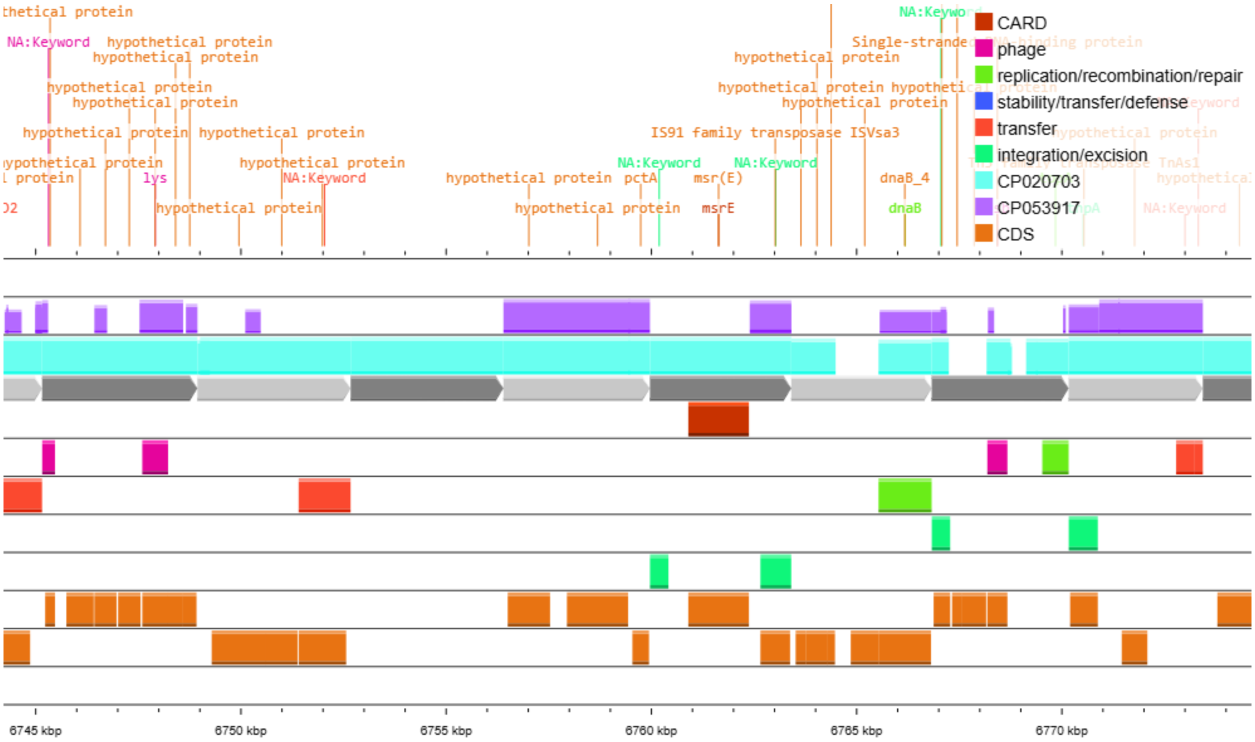

(d)

(a), (b), (c), (d). The respective focused regions of Figure 3 including identified MGEs such as prophage regions, recombination/repair protein genes, conjugative transfer genes, and integration/excision modules (Map created using https://proksee.ca/, accessed on 13 May 2025)
