# Supplementary figures and images for: Companion Animals as Reservoirs of Multidrug Resistance—A Rare Case of an XDR, NDM-1-Producing Pseudomonas aeruginosa Strain of Feline Origin in Greece
Source: Vet Sci. 2025 Jun 12;12(6):576. doi: 10.3390/vetsci12060576 (PMC12197500; doi:10.3390/vetsci12060576)

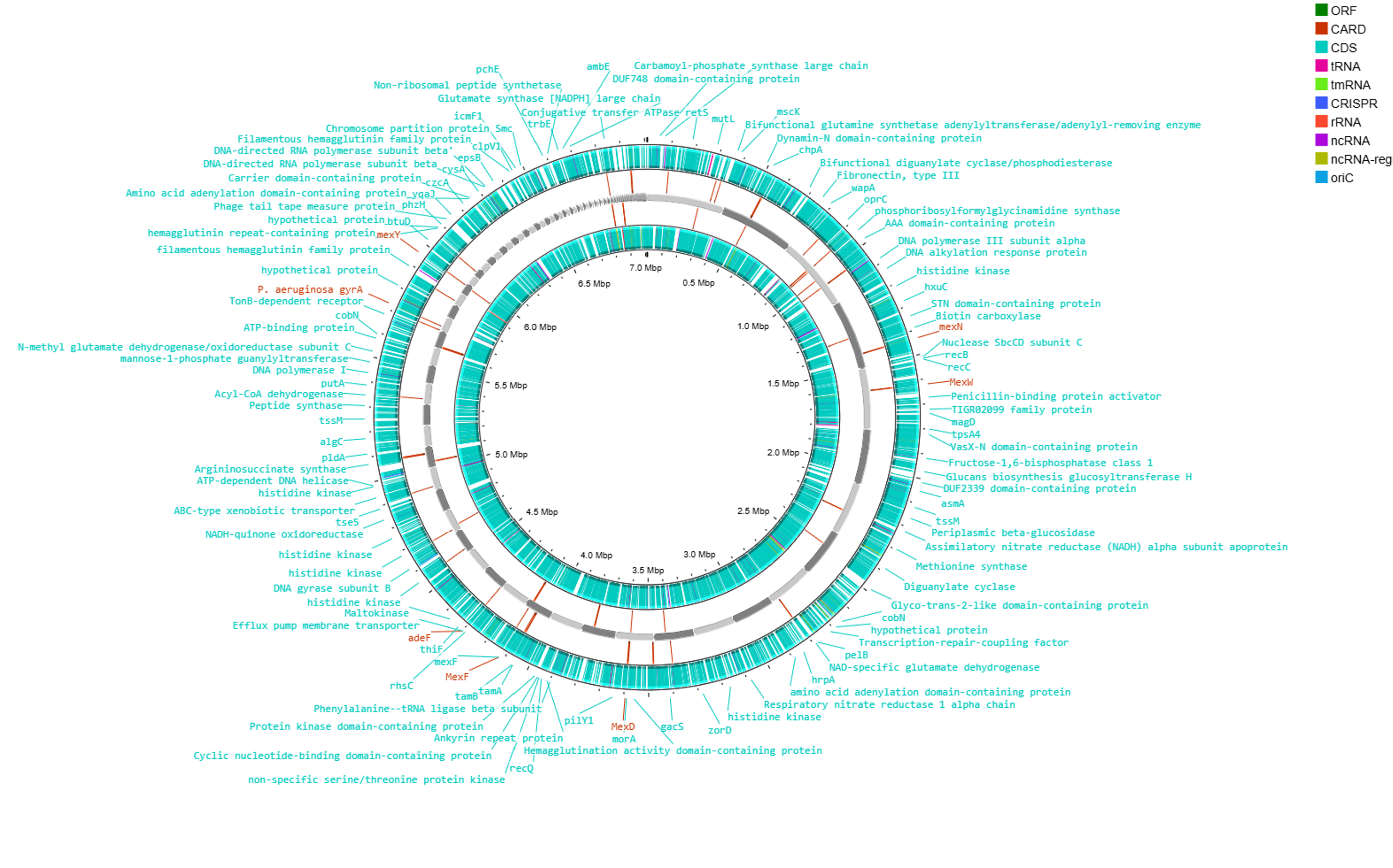

Supplement: Supplementary file 1 [file vetsci-12-00576-s001.zip › Supplementary data/Figure S2.png]
